# Pediatric tumours: liver tumours

**DOI:** 10.1186/1470-7330-14-S1-O16

**Published:** 2014-10-09

**Authors:** Alexander J Towbin

**Affiliations:** 1 Cincinnati Children’s Hospital, Department of Radiology, Cincinnati, OH 45229, USA

## 

Pediatric liver tumors account for approximately 1% of all liver tumors and up to 2% of all pediatric malignancies [1,2]. While the overall incidence of liver tumors is rare, the liver remains the third most common organ of origin for solid abdominal tumors in the pediatric population [3]. The majority of pediatric liver tumors, both benign and malignant, occur (almost) exclusively in children.

Benign tumors account for one-third of all pediatric hepatic neoplasms and include infantile hepatic hemangioma, focal nodular hyperplasia, mesenchymal hamartoma, and hepatic adenoma [2-5]. Malignant neoplasms account for the remaining two-thirds of pediatric liver tumors. The most common malignancies are hepatoblastoma, hepatocellular carcinoma, fibrolamellar hepatocellular carcinoma, undifferentiated embryonal sarcoma, and biliary rhabdomyosarcoma [4-6]. While the majority of these tumors are unique to children, focal nodular hyperplasia, hepatocellular adenomas, and hepatocellular carcinoma have counterparts in adults. The presentation, risk factors, and imaging appearance of these overlapping tumors differs in the pediatric population as compared to adults [7,8].

Even though there are a number of different liver tumors, the radiologist can often make a confident diagnosis based on the clinical information and imaging appearance of the mass [9,10]. The purpose of this lecture is to describe the imaging work-up for pediatric liver masses and then discuss each tumor, focusing on its unique clinical and imaging features.
